# Mapping Relation between Rail and Bridge Deformation Considering Nonlinear Contact of Interlayer

**DOI:** 10.3390/ma14216653

**Published:** 2021-11-04

**Authors:** Leixin Nie, Lizhong Jiang, Wangbao Zhou, Yulin Feng

**Affiliations:** 1 School of Civil Engineering, Central South University, Changsha 410075, China; nieleixing@csu.edu.cn (L.N.); lzhjiang@csu.edu.cn (L.J.); 2 National Engineering Laboratory for High-Speed Railway Construction, Central South University, Changsha 410075, China; 3 School of Civil Engineering and Architecture, East China Jiaotong University, Nanchang 330013, China; fengyulin@ecjtu.edu.cn

**Keywords:** bridge of high-speed railway, unit slab ballastless track, nonlinear contact of interlayer, mapping relationship, rail deformation, principle of stationary potential energy

## Abstract

This paper examines the effect of structural deformation on the unit slab-type ballastless track structure of high-speed railway. The principle of stationary potential energy was used to map the relation between girder vertical deformation and rail deformation considering the effect of subgrade boundary conditions and the nonlinear contact of interlayer. The theoretical model was verified by comparing with the finite element analysis and experimental results. The theoretical model was used to analyze the effects of several key parameters on the rail deformation, such as vertical deformation amplitude, elastic modulus of the mortar layer, and vertical stiffness of the fasteners. The results show that the track slabs suffered significant disengagement, which makes the deformation of the track structure at the position of the beam joint tend to be gentle when nonlinear contact between the mortar layer and the track slabs was considered. The track slabs disengagement mainly occurs near the beam joints (the side of the deformed beam). As the deflection amplitude of the girder increases, the track deformation, the fastener forces and the disengagement length of the track slabs are obviously nonlinear. When the vertical stiffness of the fastener and/or the elastic modulus of the mortar layer increase, the fastener force and the track plate disengagement length increase monotonically and nonlinearly, which will adversely affect the life and safety of the track structure.

## 1. Introduction

**Figure 1:**
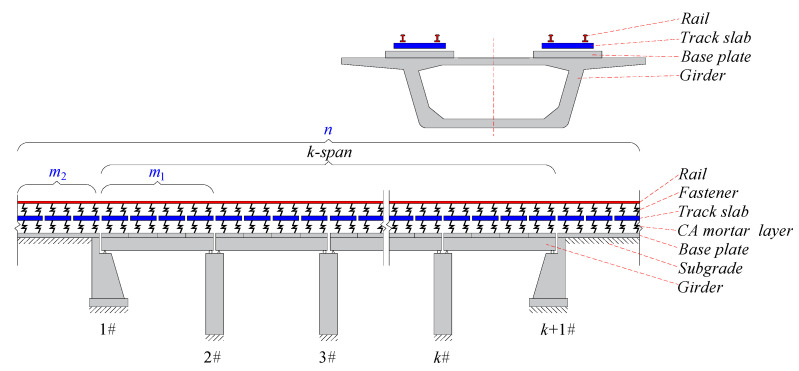
Schematic drawing of a unit slab-type ballastless track-bridge system of HSR.

At present, high-speed railway (HSR) is being rapidly developed [1,2]. In particular, ballastless track structures offer several advantages, including relatively low maintenance requirements, high strength, good stability, and high smoothness [3,4]. In the unit slab-type ballastless track [5], cement asphalt (CA) mortar forms a layer [6] between the track slabs and supporting concrete base plate as shown in Figure 1. Under multiple dynamic loading due to trains, foundation settlement, earthquakes, etc., the bridge structure would inevitably suffer from residual deformation [7,8,9,10], such as pier settlement, bearing deformation, etc. Owing to the weak contact between the mortar layer and the track slabs, bridge vertical deformation can lead to the disengagement phenomenon between the track slabs and the mortar layer. This phenomenon can cause the rail surface to suffer not only geometric irregularity but also stiffness irregularity. The irregularities of the rail can affect the comfort and safety of running trains and severely reduce the performance of the track system. Quantitative analysis of the mapping relationship between residual deformation and rail deformation is the basis of rail deformation control measures. Therefore, it is necessary to theoretically understand the mapping relationship between bridge deformation and rail geometry, especially considering the nonlinear contact between interlayers of the track structure.

To date, many studies have been performed on the track-subgrade interactions and track-bridge interactions for HSR. Some researchers [11,12,13,14,15,16,17] evaluated the mechanical properties of different track types by establishing finite element models of train-track-subgrade system and train-track-bridge system. The effects of the bridge deformations, subgrade settlement and various parameters of the bridge are also taken into account. Considering the accuracy and rationality of the finite element results, Jiang et al. [18,19,20,21,22] did lots of experiments and compared with the results of the finite element models. The problems of the track irregularities caused by structural deformations and the dynamic responses of the track structure under the action of trains were studied in detail. Their research findings provide good references for improving the performance of HSR structure.

In addition to the analysis of finite element and experimental results, other theoretical researches on the mapping relationship between track deformation and the deformation of substructures have also been carried out. Sheng et al. [23] proposed a theoretical model of the mapping relationship between track vertical deformation and ground vibration for HSR. Due to the requirement of high smoothness and the high occupation ratio of bridge in HSR, the research based on track-bridge interaction is particularly important. Chen et al. [24,25,26,27] developed an analytical solution for the mapping relation between the pier settlement of simply supported bridge and rail deflection to analyze the dynamic response of the train-track-bridge system. Aiming at the mapping relationship between the lateral and vertical deformation of the simply supported bridge and the track deformation, theoretical formulae were derived and verified through experimental results and finite element analysis [28,29]. Using an efficient method of the principle of stationary potential energy, Jiang et al. [30,31,32] derived the mapping expressions between the rail geometry and bridge deformations involving simply supported bridge structure and continuous bridge structure. The theoretical model considers the influence of subgrade boundary conditions on the deformation.

In the disengagement problem of the track structure, considering the nonlinear contacts between the subgrade and the track, a semi-analytical method of mapping relation between rail deformation and subgrade settlement was presented by Guo et al. [33]. It is shown that the unsupported regions can significantly aggravate the wheel-rail interaction. Sadeghi et al. [34] studied the dynamic influence of unsupported sleepers on rail by establishing a three-dimensional numerical model and believed that considering the sleeper-ballast contact could greatly improve the accuracy of the model calculation. Mykola et al. [35] conducted a comparative study based on numerical model and experiment, which showed that void zones in ballasted track would seriously affect the comfort and safety of train running.

However, the above theoretical methods of the mapping relation between the track geometric irregularities and the bridge deformation are almost based on elastic systems. The nonlinearity of contact between mortar layer and track slabs bottom is ignored. Based on a unit slab-type ballastless track system of HSR, this paper derived a mapping relationship between vertical bridge deformation and track geometry considering the nonlinear contact between the CA mortar layer and the track slabs. The effect of the subgrade boundary was also considered comprehensively. The accuracy of the theoretical mapping model in this paper was verified through comparing the experimental results and the finite element model (FEM) established in ANSYS. Based on this theoretical model, the effects of the amplitude of vertical bridge deformations, vertical stiffness of the fasteners, and elastic modulus of mortar layer on track geometry were analyzed quantitatively.

## 2. Theoretical Model

### 2.1. Fundamental Assumptions

A unit slab-type ballastless track-bridge system of HSR is shown in Figure 1. To establish a theoretical model for mapping the relationship between the bridge deformation and the track deformation, the following fundamental assumptions [25] are made:

(1) Theoretical model-1 (AM-1) with linear connect of interlayer
  (a) The track and girder structures are simplified as a single composite beam, in which the track slabs were vertically supported by springs (i.e., connected with the mortar layer to the base plates), regarded as free beams with two free ends, without mutual connection along the longitudinal bridge direction; the base plates and girder are firmly connected via embedded rebars. Therefore, they are assumed that deformation of the girder and base plates are synergistic;
  (b) Rail in the subgrade section can be simplified as a simply supported beam; the rail boundary effect of the subgrade section is eliminated by considering a sufficiently long section of subgrade;
  (c) The fasteners and the CA mortar layer are modeled as linear springs and uniformly distributed along the centerline of the track;
  (d) Given that the vertical bending stiffness of bridge is far greater than that of track structure, the influence of track structure on bridge deformation was ignored;
  (e) Under the action of the support displacement, the simply supported beam structure suffers vertical rigid-body deformation;
  (f) The origin of coordinates is set at the static equilibrium position of the structure under the action of gravity. Under the action of vertical deformation of the structure, the calculation is conducted on the deformation of track structure relative to the static equilibrium position of gravity. Therefore, the action of gravity was ignored in the calculations.
(2) Theoretical model-2 (AM-2) with nonlinear connect of interlayer

The CA mortar layer is considered as non-linear springs under compression only (assumption (c)). Additionally, due to the gravity of the track, there is an initial compression of the mortar layer, which affects the disengagement of the track slab. Therefore, the assumption (f) in AM-1 no longer holds true, and the weight of track structure must be taken into consideration. All other assumptions are the same as those of AM-1.

As shown in Figure 1 it was assumed that there are *k*-span simply supported beams. Additionally, there are $m_{1}$ track slabs on one-span simply supported beam and there are $m_{2}$ track slabs on the subgrade on either side of the simply supported beams.

When support displacement or rotation angle of beam-end occurs to the simply supported beam, the function for the girder deflection can be expressed as:

(1) $$y_{i}=a_{i}x_{i}+b_{i}$$

where $a_{i}$ and $b_{i}$ are constant coefficients, and thereinto $i\in \left[m_{2}+1,\;m_{2}+km_{1}\right]$.

### 2.2. Establishment of Theoretical Model for Deformation Mapping Relationship

#### 2.2.1. Theoretical Model of Nonlinear Connect of Interlayer without Track Slab Disengagement

Bending strain energy and gravitational potential energy of rail and track slabs are expressed as:

(2) $$U=\sum_{i=1}^{n}\left[\int_{0}^{L_{i}}\left(\frac{1}{2}E_{1}I_{1}u_{i}{}^{\prime \prime }{}^{2}+\frac{1}{2}E_{2}I_{2}v_{i}{}^{\prime \prime }{}^{2}\right)dx-\int_{0}^{L_{i}}\left(\rho_{1}gu_{i}+\rho_{2}gv_{i}\right)dx\right]$$

where $E_{1}I_{1}$ and $E_{2}I_{2}$ denote the vertical bending stiffness of rail and track slab, respectively. $u_{i}$ and $v_{i}$ denote the deflection of rail and track slab, respectively. $L_{i}$ denotes the length of the $i^{th}$ track slab and n denotes the total number of track slabs. $\rho_{1}$ and $\rho_{2}$ denote the linear density of rail and track slab, respectively. g is the acceleration of gravity.

The deformation energy of fasteners and CA mortar layer is:

(3) $$V=\sum_{i=1}^{n}\int_{0}^{L_{i}}\left(\frac{1}{2}k_{1}{\left(u_{i}-v_{i}\right)}^{2}+\frac{1}{2}k_{2}{\left(v_{i}-y_{i}\right)}^{2}\right)dx$$

where $k_{1}$ and $k_{2}$ denote the vertical equivalent stiffness of fasteners and CA mortar layer, respectively.

The system’s total potential energy is expressed as:

(4) $$\begin{array}{cc} \Pi & =U+V\\ & =\sum_{i=1}^{n}\int_{0}^{L_{i}}\left[\begin{array}{l} \frac{1}{2}E_{1}I_{1}u_{i}{{}^{\prime \prime }}^{2}+\frac{1}{2}E_{2}I_{2}v_{i}{{}^{\prime \prime }}^{2}+\frac{1}{2}k_{1}{\left(u_{i}-v_{i}\right)}^{2}\\ +\frac{1}{2}k_{2}{\left(v_{i}-y_{i}\right)}^{2}-\rho_{1}gu_{i}-\rho_{2}gv_{i} \end{array}\right]dx \end{array}$$

Based on the principle of stationary potential energy, first variation was taken for the total potential energy of the system and the following was obtained:

(5) $$\delta \Pi =\sum_{i=1}^{n}\int_{0}^{L_{i}}\left[\begin{array}{l} E_{1}I_{1}u_{i}{}^{\prime \prime }\delta u_{i}{}^{\prime \prime }+E_{2}I_{2}v_{i}{}^{\prime \prime }\delta v_{i}{}^{\prime \prime }\\ +k_{1}\left(u_{i}-v_{i}\right)\delta u_{i}-k_{1}\left(u_{i}-v_{i}\right)\delta v_{i}+k_{2}\left(v_{i}-y_{i}\right)\delta v_{i}\\ -\rho_{1}g\delta u_{i}-\rho_{2}g\delta v_{i} \end{array}\right]dx$$

Through integration by parts, the above formula was transformed into:

(6) $$\delta \Pi =\sum_{i=1}^{n}\left\{\begin{array}{l} \left({E_{1}I_{1}u_{i}{}^{\prime \prime }\delta u_{i}{}^{\prime }|}_{0}^{L_{i}}-{E_{1}I_{1}u_{i}{}^{\prime \prime \prime }\delta u_{i}|}_{0}^{L_{i}}+{E_{2}I_{2}v_{i}{}^{\prime \prime }\delta v_{i}{}^{\prime }|}_{0}^{L_{i}}-{E_{2}I_{2}v_{i}{}^{\prime \prime \prime }\delta v_{i}|}_{0}^{L_{i}}\right)\\ +\int_{0}^{L_{i}}\left[\begin{array}{l} \left(E_{1}I_{1}u_{i}{}^{\left(4\right)}+k_{1}\left(u_{i}-v_{i}\right)-\rho_{1}g\right)\delta u_{i}\\ +\left(E_{2}I_{2}v_{i}{}^{\left(4\right)}-k_{1}\left(u_{i}-v_{i}\right)+k_{2}\left(v_{i}-y_{i}\right)-\rho_{2}g\right)\delta v_{i} \end{array}\right]dx \end{array}\right\}$$

According to Equation (6), the equilibrium differential equation and natural boundary conditions for vertical deformation of bridge-track system could be obtained as follows:

(7) $$E_{1}I_{1}u_{i}{}^{\left(4\right)}+k_{1}\left(u_{i}-v_{i}\right)-\rho_{1}g=0$$

(8) $$E_{2}I_{2}v_{i}{}^{\left(4\right)}-k_{1}\left(u_{i}-v_{i}\right)+k_{2}\left(v_{i}-y_{i}\right)-\rho_{2}g=0$$

(9) $$\begin{array}{l} {v_{i}{}^{\prime \prime }|}_{x=0}=0\;{v_{i}{}^{\prime \prime }|}_{x=L_{i}}=0\\ {v_{i}{}^{\prime \prime \prime }|}_{x=0}=0\;{v_{i}{}^{\prime \prime \prime }|}_{x=L_{i}}=0 \end{array}$$

(10) $$\left\{ \begin{array}{l} {u_{1}|}_{x=0}={u_{1}{}^{\prime \prime }|}_{x=0}=0\;{\;u_{n}|}_{x=L_{n}}={u_{n}{}^{\prime \prime }|}_{x=L_{n}}=0\\ {\;u_{i}|}_{x=L_{i}}={u_{i+1}|}_{x=0}\;{u_{i}{}^{\prime }|}_{x=L_{i}}={u_{i+1}{}^{\prime }|}_{x=0}\\ {u_{i}{}^{\prime \prime }|}_{x=L_{i}}={u_{i+1}{}^{\prime \prime }|}_{x=0}\;{u_{i}{}^{\prime \prime \prime }|}_{x=L_{i}}={u_{i+1}{}^{\prime \prime \prime }|}_{x=0}\; \end{array}\right.$$

where i=1, 2, …, n.

Using Equations (7) and (8), the following expression was obtained:

(11) $$\begin{array}{l} \frac{E_{1}I_{1}E_{2}I_{2}}{k_{1}}u_{i}{}^{\left(8\right)}+\left(E_{1}I_{1}+E_{2}I_{2}+\frac{E_{1}I_{1}k_{2}}{k_{1}}\right)u_{i}{}^{\left(4\right)}+k_{2}u_{i}\\ =k_{2}y_{i}+\left(1+\frac{k_{2}}{k_{1}}\right)\rho_{1}g+\rho_{2}g \end{array}$$

Assuming $D=\frac{d}{dx}$ and substituting it into Equation (11), the following expression was obtained:

(12) $$\begin{array}{l} \left[\frac{E_{1}I_{1}E_{2}I_{2}}{k_{1}}D^{\left(8\right)}+\left(E_{1}I_{1}+E_{2}I_{2}+\frac{E_{1}I_{1}k_{2}}{k_{1}}\right)D^{\left(4\right)}+k_{2}\right]u_{i}\\ =k_{2}y_{i}+\left(1+\frac{k_{2}}{k_{1}}\right)\rho_{1}g+\rho_{2}g \end{array}$$

The characteristic equation for Equation (12) is:

(13) $$\frac{E_{1}I_{1}E_{2}I_{2}}{k_{1}}\lambda^{8}+\left(E_{1}I_{1}+E_{2}I_{2}+\frac{E_{1}I_{1}k_{2}}{k_{1}}\right)\lambda^{4}+k_{2}=0$$

Assuming $\alpha =\frac{k_{1}}{E_{1}I_{1}}+\frac{k_{1}}{E_{2}I_{2}}+\frac{k_{2}}{E_{2}I_{2}}$, $\beta =\frac{k_{1}k_{2}}{E_{1}I_{1}E_{2}I_{2}}$, and the following was obtained:

(14) $${\left(\lambda^{4}+\frac{\alpha }{2}\right)}^{2}-\left(\frac{\alpha^{2}}{4}-\beta \right)=0$$

where $\frac{\alpha^{2}}{4}-\beta =\frac{1}{4}{\left(\frac{k_{1}}{E_{1}I_{1}}+\frac{k_{1}}{E_{2}I_{2}}+\frac{k_{2}}{E_{2}I_{2}}\right)}^{2}-\frac{k_{1}k_{2}}{E_{1}I_{1}E_{2}I_{2}}>\frac{1}{4}\left[{\left(\frac{k_{1}}{E_{1}I_{1}}+\frac{k_{2}}{E_{2}I_{2}}\right)}^{2}-\frac{4k_{1}k_{2}}{E_{1}I_{1}E_{2}I_{2}}\right]\geq 0$.

Assuming $\eta^{2}=\frac{\alpha^{2}}{4}-\beta$, and the following was obtained:

(15) $$\left(\lambda^{4}+\frac{\alpha }{2}+\eta \right)\left(\lambda^{4}+\frac{\alpha }{2}-\eta \right)=0$$

The solution of the characteristic equation for Equation (12) can be expressed as:

(16) $$\left\{ \begin{array}{l} \lambda_{1,2,3,4}=\pm N_{1}\pm iN_{1}\\ \lambda_{5,6,7,8}=\pm N_{2}\pm iN_{2} \end{array}\right.$$

where $4N_{1}{}^{4}=\frac{\alpha }{2}+\eta >0$, $4N_{2}{}^{4}=\frac{\alpha }{2}-\eta >0$, $N_{1}=\sqrt[{4}]{\frac{\alpha }{8}+\frac{\eta }{4}}$, $N_{2}=\sqrt[{4}]{\frac{\alpha }{8}-\frac{\eta }{4}}$.

The homogeneous solution for Equation (12) can be expressed as:

(17) $$\begin{array}{cc} {\bar{u}}_{i} & =e^{-N_{1}x}\left(A_{i}cosN_{1}x+B_{i}sinN_{1}x\right)+e^{N_{1}x}\left(C_{i}cosN_{1}x+D_{i}sinN_{1}x\right)\\ & +e^{N_{2}x}\left(E_{i}cosN_{2}x+F_{i}sinN_{2}x\right)+e^{-N_{2}x}\left(G_{i}cosN_{2}x+H_{i}sinN_{2}x\right) \end{array}$$

where $A_{i}$, $B_{i}$, $C_{i}$, $D_{i}$, $E_{i}$, $F_{i}$, $G_{i}$ and $H_{i}$ denote the coefficients to be calculated, and thereinto, i=1, 2, …, n.

Given the function of Equation (1) for the girder deflection, the particular solution of Equation (12) can be expressed as:

(18) $${\tilde{u}}_{i}=y_{i}+\left(\frac{1}{k_{1}}+\frac{1}{k_{2}}\right)\rho_{1}g+\frac{1}{k_{2}}\rho_{2}g$$

Therefore, the general solution of Equation (12) can be expressed as:

(19) $$\begin{array}{cc} u_{i} & =e^{-N_{1}x}\left(A_{i}cosN_{1}x+B_{i}sinN_{1}x\right)+e^{N_{1}x}\left(C_{i}cosN_{1}x+D_{i}sinN_{1}x\right)\\ & +e^{N_{2}x}\left(E_{i}cosN_{2}x+F_{i}sinN_{2}x\right)+e^{-N_{2}x}\left(G_{i}cosN_{2}x+H_{i}sinN_{2}x\right)\\ & +y_{i}+\left(\frac{1}{k_{1}}+\frac{1}{k_{2}}\right)\rho_{1}g+\frac{\rho_{2}g}{k_{2}} \end{array}$$

Substituting Equation (19) into Equation (7), the deflection of track slabs can be expressed as:

(20) $$\begin{array}{cc} v_{i} & =X_{1}\left[e^{-N_{1}x}\left(A_{i}cosN_{1}x+B_{i}sinN_{1}x\right)+e^{N_{1}x}\left(C_{i}cosN_{1}x+D_{i}sinN_{1}x\right)\right]\\ & +X_{2}\left[e^{N_{2}x}\left(E_{i}cosN_{2}x+F_{i}sinN_{2}x\right)+e^{-N_{2}x}\left(G_{i}cosN_{2}x+H_{i}sinN_{2}x\right)\right]\\ & +y_{i}+\frac{\rho_{1}g+\rho_{2}g}{k_{2}} \end{array}$$

where $X_{1}=\left(-\frac{4E_{1}I_{1}}{k_{1}}N_{1}{}^{4}+1\right)$, $X_{2}=\left(-\frac{4E_{1}I_{1}}{k_{1}}N_{2}{}^{4}+1\right)$

Substituting Equations (19) and (20) into Equations (9) and (10), a total of 8*n* algebraic equations were obtained. The coefficients $A_{i}$, $B_{i}$, $C_{i}$, $D_{i}$, $E_{i}$, $F_{i}$, $G_{i}$ and $H_{i}$ were easily calculated, where i=1, 2, …, n. Substituting them into Equations (19) and (20), the analytical expression for rail deflection $u_{i}$ and track slabs deflection $v_{i}$ were obtained.

#### 2.2.2. Theoretical Model of Nonlinear Connect of Interlayer with Track Slab Disengagement

**Figure 2:**
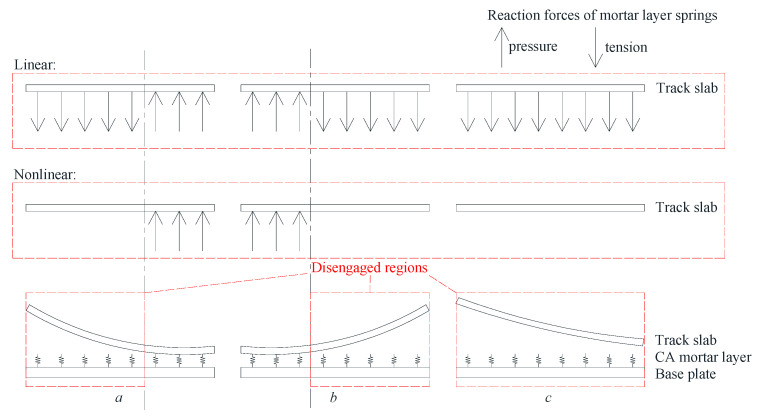
Schematic drawing of track slab disengagement and the reaction forces of mortar layer to the track slabs (**a**), and (**b**) partial track slab disengagement; (**c**) full track slab disengagement.

As is shown in Figure 2, when deformation of the track slab is smaller than deformation of the base plate (girder), partial disengagement occurs between the track slab and CA mortar layer. Thus, stress in mortar layer is zero and $k_{2}=0$. Assuming the track slab disengagement occurs in the $i^{th}$ section and the disengagement length is $L_{i}$, Equation (3) becomes:

(21) $$V=\sum_{i=1}^{n}\int_{0}^{L_{i}}\left(\frac{1}{2}k_{1}{\left(u_{i}-v_{i}\right)}^{2}\right)dx$$

The equilibrium equation of vertical deformation of the bridge-track system in the section of the track slab with disengagement is

(22) $$E_{1}I_{1}u_{i}{}^{\left(4\right)}+k_{1}\left(u_{i}-v_{i}\right)-\rho_{1}g=0$$

(23) $$E_{2}I_{2}v_{i}{}^{\left(4\right)}-k_{1}\left(u_{i}-v_{i}\right)-\rho_{2}g=0$$

According to Figure 2, different cases of disengagement correspond to different boundary conditions of the track slabs.

For the case presented in Figure 2a:

(24) $$\left\{ \begin{array}{ll} {v_{i}|}_{x=L_{i}}={v_{i+1}|}_{x=0} & \;{v_{i}{}^{\prime }|}_{x=L_{i}}={v_{i+1}{}^{\prime }|}_{x=0}\\ {v_{i}{}^{\prime \prime }|}_{x=L_{i}}={v_{i+1}{}^{\prime \prime }|}_{x=0} & \;{v_{i}{}^{\prime \prime \prime }|}_{x=L_{i}}={v_{i+1}{}^{\prime \prime \prime }|}_{x=0} \end{array}\right.$$

For the case presented in Figure 2b:

(25) $$\left\{ \begin{array}{ll} {v_{i-1}|}_{x=L_{i}}={v_{i}|}_{x=0} & \;{v_{i-1}{}^{\prime }|}_{x=L_{i}}={v_{i}{}^{\prime }|}_{x=0}\;\\ {v_{i-1}{}^{\prime \prime }|}_{x=L_{i}}={v_{i}{}^{\prime \prime }|}_{x=0} & \;{v_{i-1}{}^{\prime \prime \prime }|}_{x=L_{i}}={v_{i}{}^{\prime \prime \prime }|}_{x=0} \end{array}\right.$$

For the case presented in Figure 2c:

(26) $$\left\{ \begin{array}{ll} {v_{i}{}^{\prime \prime }|}_{x=0}=0 & \;{v_{i}{}^{\prime \prime }|}_{x=L_{i}}=0\\ {v_{i}{}^{\prime \prime \prime }|}_{x=0}=0 & \;{v_{i}{}^{\prime \prime \prime }|}_{x=L_{i}}=0 \end{array}\right.$$

Boundary conditions of the rail are:

(27) $$\left\{ \begin{array}{ll} {u_{1}|}_{x=0}={u_{1}{}^{\prime \prime }|}_{x=0}=0 & \;{\;u_{n}|}_{x=L_{n}}={u_{n}{}^{\prime \prime }|}_{x=L_{n}}=0\\ {u_{i}|}_{x=L_{i}}={u_{i+1}|}_{x=0} & \;{u_{i}{}^{\prime }|}_{x=L_{i}}={u_{i+1}{}^{\prime }|}_{x=0}\\ {u_{i}{}^{\prime \prime }|}_{x=L_{i}}={u_{i+1}{}^{\prime \prime }|}_{x=0} & \;{u_{i}{}^{\prime \prime \prime }|}_{x=L_{i}}={u_{i+1}{}^{\prime \prime \prime }|}_{x=0}\; \end{array}\right.$$

Using Equations (22) and (23), the following expression was obtained:

(28) $$\frac{E_{1}I_{1}E_{2}I_{2}}{k_{1}}u_{i}{}^{\left(8\right)}+\left(E_{1}I_{1}+E_{2}I_{2}\right)u_{i}{}^{\left(4\right)}=\rho_{1}g+\rho_{2}g$$

Assuming $D=\frac{d}{dx}$ and substituting it into Equation (28), the following expression was obtained:

(29) $$\left[\frac{E_{1}I_{1}E_{2}I_{2}}{k_{1}}D^{\left(8\right)}+\left(E_{1}I_{1}+E_{2}I_{2}\right)D^{\left(4\right)}\right]u_{i}=\rho_{1}g+\rho_{2}g$$

The characteristic equation for Equation (29) is:

(30) $$\frac{E_{1}I_{1}E_{2}I_{2}}{k_{1}}\lambda^{8}+\left(E_{1}I_{1}+E_{2}I_{2}\right)\lambda^{4}=0$$

Assuming $\alpha =\frac{k_{1}}{E_{1}I_{1}}+\frac{k_{1}}{E_{2}I_{2}}$, and the following was obtained:

(31) $$\lambda^{4}\left(\lambda^{4}+\alpha \right)=0$$

The solution of the characteristic equation for Equation (30) can be expressed as:

(32) $$\left\{ \begin{array}{l} \lambda_{1,2,3,4}=\pm N_{1}\pm iN_{1}\\ \lambda_{5,6,7,8}=0 \end{array}\right.$$

where $4N_{1}{}^{4}=\alpha >0$, $N_{1}=\sqrt[{4}]{\frac{\alpha }{4}}$.

The homogeneous equation for Equation (30) can be expressed as:

(33) $$\begin{array}{cc} {\bar{u}}_{i} & =e^{-N_{1}x}\left(A_{i}cosN_{1}x+B_{i}sinN_{1}x\right)+e^{N_{1}x}\left(C_{i}cosN_{1}x+D_{i}sinN_{1}x\right)\\ & +E_{i}+F_{i}x+G_{i}x^{2}+H_{i}x^{3} \end{array}$$

where $A_{i}$, $B_{i}$, $C_{i}$, $D_{i}$, $E_{i}$, $F_{i}$, $G_{i}$ and $H_{i}$ denote the coefficient to be calculated, and thereinto, i=1, 2, …, n.

The particular solution of Equation (29) can be expressed as:

(34) $${\tilde{u}}_{i}=\frac{\rho_{1}g+\rho_{2}g}{24\left(E_{1}I_{1}+E_{2}I_{2}\right)}x^{4}$$

Therefore, the general solution of Equation (29) for the unsupported slabs can be expressed as:

(35) $$\begin{array}{cc} u_{i} & =e^{-N_{1}x}\left(A_{i}cosN_{1}x+B_{i}sinN_{1}x\right)+e^{N_{1}x}\left(C_{i}cosN_{1}x+D_{i}sinN_{1}x\right)\\ & +E_{i}+F_{i}x+G_{i}x^{2}+H_{i}x^{3}+\frac{\rho_{1}g+\rho_{2}g}{24\left(E_{1}I_{1}+E_{2}I_{2}\right)}x^{4} \end{array}$$

Substituting Equation (35) into Equation (22), the deflection of track slab for the unsupported slabs can be expressed as:

(36) $$\begin{array}{cc} v_{i} & =X_{1}\left[e^{-N_{1}x}\left(A_{i}cosN_{1}x+B_{i}sinN_{1}x\right)+e^{N_{1}x}\left(C_{i}cosN_{1}x+D_{i}sinN_{1}x\right)\right]\\ & +E_{i}+F_{i}x+G_{i}x^{2}+H_{i}x^{3}+\frac{\rho_{1}g+\rho_{2}g}{E_{1}I_{1}+E_{2}I_{2}}\left(\frac{x^{4}}{24}+\frac{E_{1}I_{1}}{k_{1}}\right)-\frac{\rho_{1}g}{k_{1}} \end{array}$$

#### 2.2.3. Theoretical Model of Linear Connect of Interlayer 

When the theoretical model is linear connect of interlayer, the gravitational acceleration is zero (assumption (f) in AM-1). The rail deflection between the track slabs and CA mortar layer can be obtained as follows [31]:

(37) $$\begin{array}{cc} u_{i} & =e^{-N_{1}x}\left(A_{i}cosN_{1}x+B_{i}sinN_{1}x\right)+e^{N_{1}x}\left(C_{i}cosN_{1}x+D_{i}sinN_{1}x\right)\\ & +e^{N_{2}x}\left(E_{i}cosN_{2}x+F_{i}sinN_{2}x\right)+e^{-N_{2}x}\left(G_{i}cosN_{2}x+H_{i}sinN_{2}x\right)+y_{i} \end{array}$$

Similarly, the deflection of track slab can be expressed as:

(38) $$\begin{array}{cc} v_{i} & =X_{1}\left[e^{-N_{1}x}\left(A_{i}cosN_{1}x+B_{i}sinN_{1}x\right)+e^{N_{1}x}\left(C_{i}cosN_{1}x+D_{i}sinN_{1}x\right)\right]\\ & +X_{2}\left[e^{N_{2}x}\left(E_{i}cosN_{2}x+F_{i}sinN_{2}x\right)+e^{-N_{2}x}\left(G_{i}cosN_{2}x+H_{i}sinN_{2}x\right)\right]+y_{i} \end{array}$$

### 2.3. Theoretical Solution to Mapping Relationship

**Figure 3:**
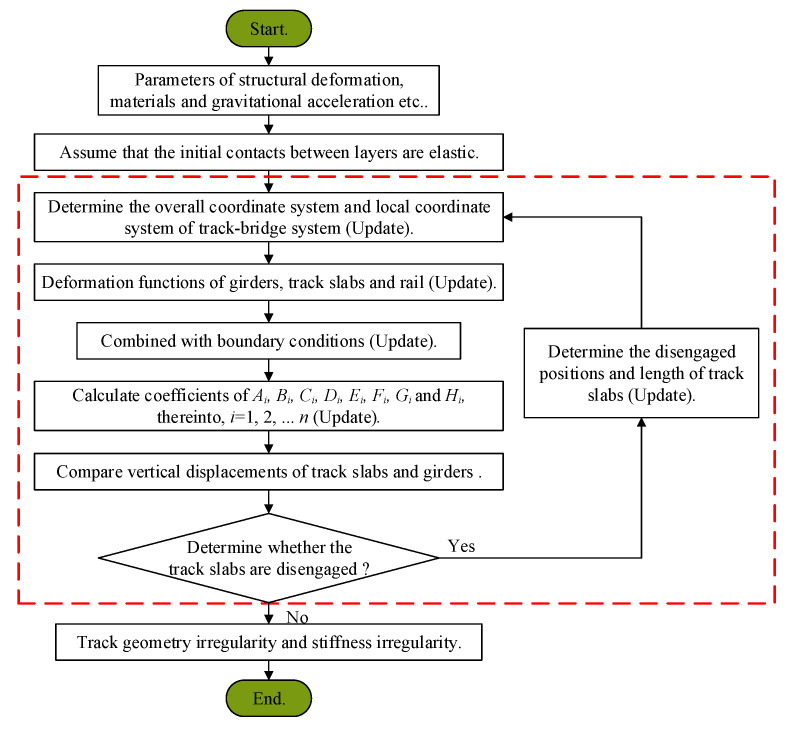
Flowchart of calculation process for AM-2.

When the effect of track slabs disengagement is considered, the contact relationship is non-linear. The structure is statically indeterminate, therefore, the equilibrium equations are insufficient. In this paper, successive approximation is used to solve the problem, as follows: (1) assume there are good contacts between the track slabs and the CA mortar layer at the initial time, then solve the vertical displacement of ballastless track using the method described in Section 2.2.1; (2) compare the vertical displacement of the track slab with the displacement of the base plate to assess whether the track slabs suffer from disengagement and, if so, determine the locations and disengagement lengths; (3) update the contact status of track slabs and solve the vertical displacement of ballastless track using the method described in Section 2.2.2; (4) compare the vertical displacement of the track slabs with the displacement of the base plates, then update the locations and disengagement lengths; (5) repeat Steps (2)–(4) until the contact status of the track slabs no longer changes. Then, stop the iteration as the solution has been found. A flowchart of the calculation process is presented in Figure 3.

## 3. Verification of Theoretical Model

### 3.1. Finite Element Model

AM-2 can take into account the effect of the track slabs disengagement on rail mapping deformation. To verify the correctness of the AM-2, a finite element model (FEM) of the unit slab-type ballastless track-bridge system of HSR was established in ANSYS. The 3D element BEAM188 was used for the rail, track slab, base plate, girder and pier; the linear spring element COMBIN14 was used for the fasteners. The fasteners are arranged along the center line of the rails according to the actual fastener spacing; the CA mortar layer can only withstand the pressure, so the nonlinear spring element COMBIN40 was used for the CA mortar layer. The springs of the CA mortar layer are arranged along the center line of the track slabs according to the 1/3 length of the fastener spacing; the connections between the master and slave nodes used the MPC184 rigid beam element to simulate. Deformation of the girder structure was simulated by applying displacement constraints at the bearings and piers’ bottom. The boundary effect in the subgrade section underneath the rail was eliminated by considering a sufficiently long section of subgrade.

**Figure 4:**
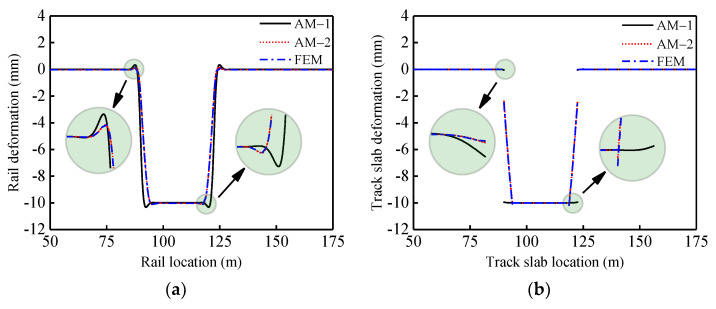
Case 1 (**a**) rail deformation; (**b**) track slab deformation.

**Figure 5:**
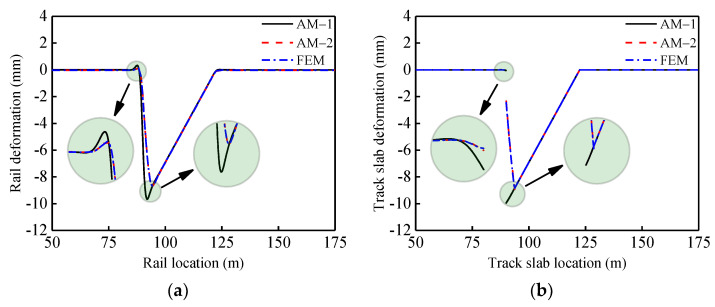
Case 2 (**a**) rail deformation; (**b**) track slab deformation.

To verify the theoretical calculation method, the unit slab-type ballastless track-bridge system of a HSR was used as an example: subgrade (150 m) + simply-supported beams (4 × 32.5 m^2^) + subgrade (150 m). Specific dimensions and physical parameters of AM and FEM are presented in Table 1. The FEM and the theoretical method were used to perform the rail deformation mapping in two typical cases: simply-supported beam vertical fault of 10 mm (Case 1) and vertical deformation of 10 mm at the girder end (Case 2). The results are presented in Figure 4 and Figure 5.

According to Figure 4 and Figure 5 and Table 2, when the girder is vertically deformed by 10 mm, the deformation will also occur in the upper track system. Within the regions entering and leaving the deformation area of the girder, the rail will undergo varying degrees of convex deformation. In the two cases, values of rail vertical deformation were calculated using AM-1, AM-2 and FEM. Comparing models AM-2 and FEM, differences were not greater than 5%, which verify the accuracy of AM-2. When the nonlinear connection of interlayers (CA mortar layer) was considered, the track slabs suffered significant disengagement in both two cases. Comparing with AM-1, the results of AM-2 and FEM, the values at the junction of concavities and convexities of the rail decreased significantly on the inside of the beam joints or on the outside. Owing to the disengagement of the track slabs, the stiffness irregularity occurred at the same time as the geometric irregularity of the track structure. The performance of the track system was significantly reduced.

### 3.2. Experimental Verification

**Figure 6:**
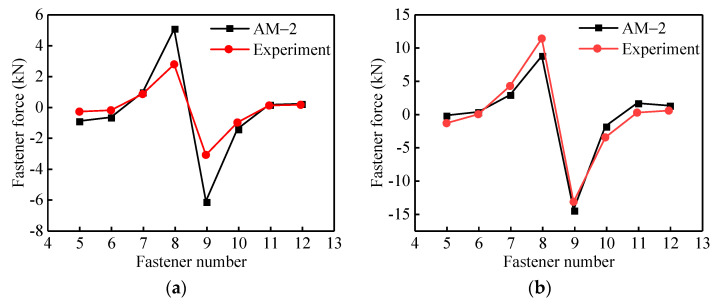
Comparison of theoretical results and experimental results of fastener forces: (**a**) 1# girder vertical fault 0.5 mm; (**b**) 1# girder vertical fault 2.5 mm.

**Figure 7:**
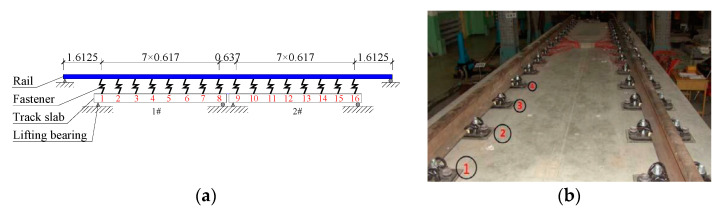
Schematic diagram of experimental model (unit: m): (**a**) Schematic diagram of fastener arrangement; (**b**) Photography of the experimental model.

In order to further verify the rationality and accuracy of the theoretical model in this paper, the theoretical model AM-2 considering the nonlinear contact between layers is compared with the measured fastener force in the test conducted by Wei [36], as shown in Figure 6. The experimental model is shown in Figure 7. 

Figure 6 shows the force distribution of no. 5 to No. 12 fasteners when 1# girder fault is 0.5 mm and 2.5 mm. The results of theoretical model AM-2 were compared with experimental results [36]. The comparison results show that the variation law of fastener additional forces in the model AM-2 of this paper is the same as the measured results. The fastener additional forces on both sides of the beam joint are anti-symmetric, and the fasteners on one side are tensioned and the other side are compressed. It can be seen from Figure 6a that the theoretical values in this paper are greater than the measured values under the working condition of the 0.5 mm girder fault. The main reasons are that there are fastener installation gaps in the test model, which causes larger calculating errors when the girder fault is smaller. The calculation error causing by installation will decrease with increasing girder fault, as shown in Figure 6b.

## 4. The Effect of Deformation Amplitude of Bridge Structure on Track Deformation

**Figure 8:**
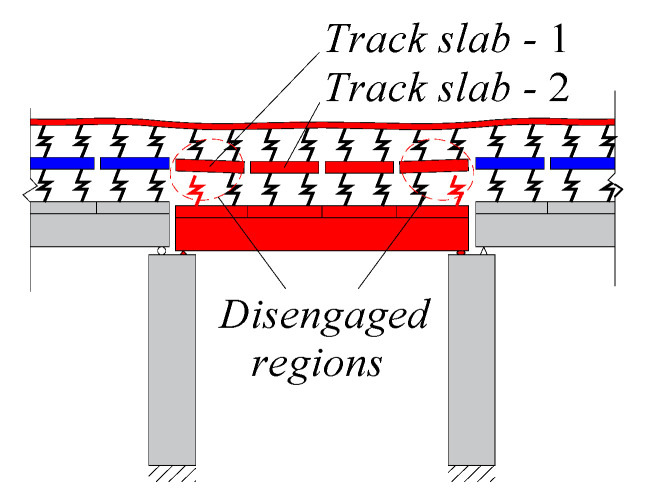
Schematic diagram of girder vertical fault.

To study the effect of the vertical deformation amplitude of the bridge structure on the rail deformation and interlayer contact, AM-2 was used to analyze the track deformation for six amplitudes of girder vertical fault (see Figure 8): 0.5 mm, 1 mm, 3 mm, 5 mm, 10 mm, 15 mm.

### 4.1. The Effect of Bridge Structure Deformation Amplitude on Track Deformation

**Figure 9:**
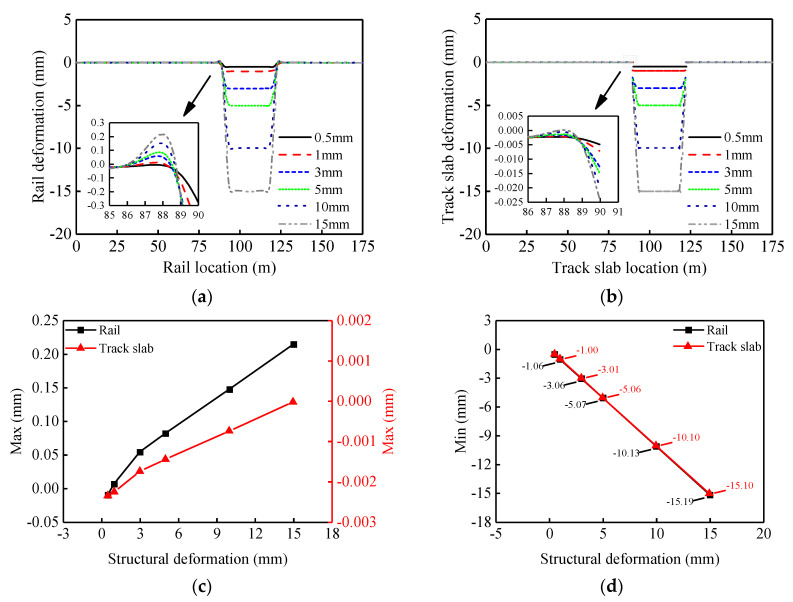
Variation of track vertical deformation with girder vertical fault: (**a**) vertical rail deformation; (**b**) vertical track slab deformation; (**c**) upward deformation of rail and track slab; (**d**) downward deformation of rail and track slab.

Figure 9a,b shows the rail deformation and track slabs deformation for different amplitudes of girder vertical fault. When the girder vertical fault occurs, the rail and track slabs undergo corresponding deformation and the amplitude of the mapping deformation will increase significantly with increasing deflection of the girder. On the outer side of the beam joint (settlement region was set as the inner side), both the rail and track slabs undergo slight convex deformation. On the inside of the beam joint, the rail undergoes slight concave deformation, while the track slabs warp upwards and suffer significant disengagement (see Figure 9b). Figure 9c shows that as the deflection amplitude of the girder increases, maximum convex deformations of the rail and the track slabs occur on the outside of the beam joint. Both of the increases in deformation is not strictly linear. There is a nearly ten-fold decrease in maximum deformation of the track slab. As shown in Figure 9d, as the amplitude of deflection of the girder increases, concave deformations of the rail and track slabs both increase linearly, with approximately the same amount of deformation.

### 4.2. The Effect of Bridge Structure Deformation Amplitude on the Interlayer Contact of Track Structure

**Figure 10:**
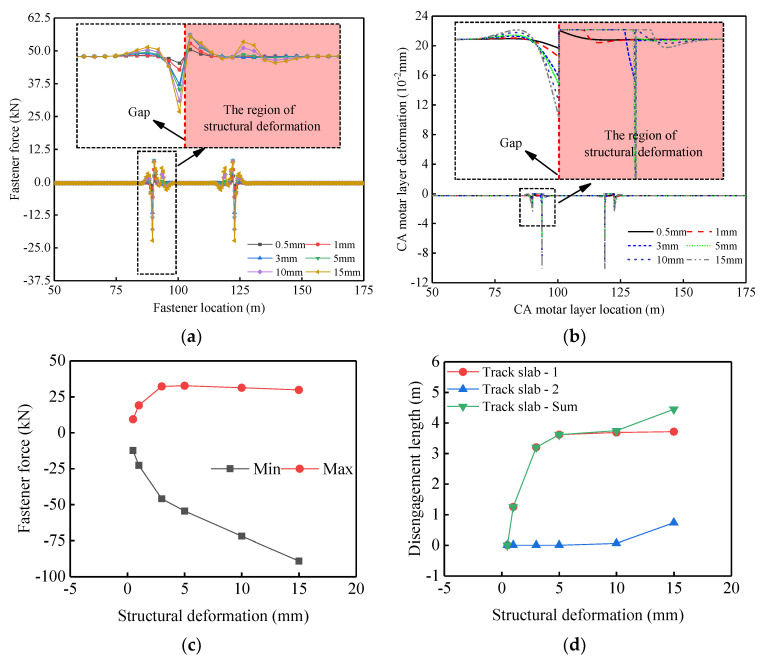
Variation of interlayer contact characteristics of track structure with girder vertical fault: (**a**) variation of fastener forces distribution; (**b**) variation of CA mortar layer deformation distribution; (**c**) variation of fastener force extremum; (**d**) variation of track slab disengagement length.

When the girder vertical fault occurs, the distributions of vertical fastener forces and vertical mortar layer deformation are both asymmetrical along the mid-span of the girder, as illustrated in Figure 10a,b. According to Figure 10b, when the pull force of the fasteners is greater than the self-gravity of the track slabs, the track slabs will be lifted and the mortar layer deformation will recover. As the deflection amplitude of the girder increases, the fastener forces and the CA mortar layer compression continue to increase and the disengagement of the track slab worsens. Disengagement first occurs on track slab-1 next to the inner side of the beam joint (see Figure 8). When the value of girder vertical fault exceeds 5 mm, the disengagement rapidly extends across the entire track slab, then extends to the neighboring track slab-2, as per Figure 10d. As shown in Figure 10c, the increase of fastener force is non-linear. The maximum tension on the fastener becomes stable when the value of girder vertical fault exceeds 3 mm. The variation is primarily that upon the disengagement of track slab, the interaction decreases between the bridge structure and track slabs. In this situation, the resultant force acting on the fasteners is mainly comprised of the self-weight of the track slabs.

## 5. The Effects of Interlayer Characteristics of the Track Structure on Track Deformation

### 5.1. The Effect of the Vertical Stiffness of Fasteners on Track Deformation

**Figure 11:**
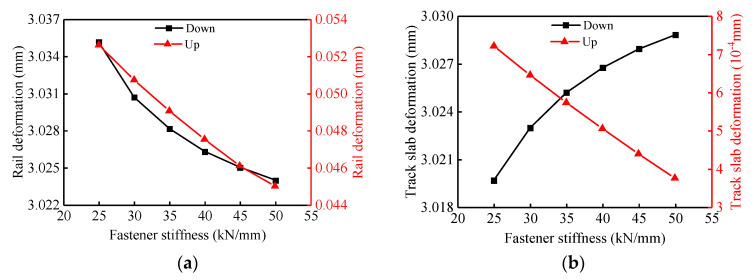

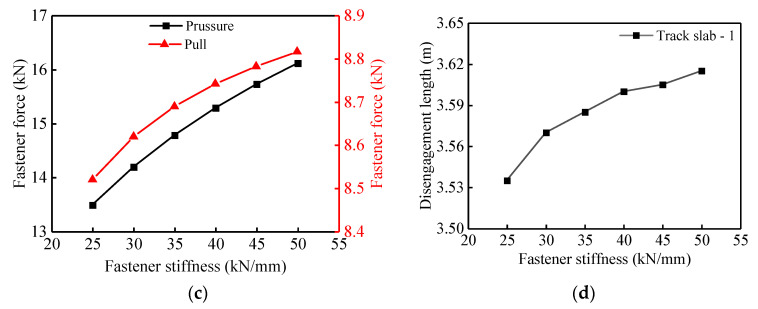
Effects of different fastener vertical stiffness on track structures: (**a**) variation of rail deformation; (**b**) variation of track slab deformation; (**c**) variation of fastener force extremum; (**d**) variation of track slab disengagement length.

AM-2 was used to calculate the vertical deformations of rail and track slabs, fastener force, and disengagement length of the track slabs when the girder undergoes a vertical fault of 3 mm. Six cases of vertical stiffness of the fasteners were considered: 25 kN/mm, 30 kN/mm, 35 kN/mm, 40 kN/mm, 45 kN/mm, and 50 kN/mm. The results are presented in Figure 11.

According to the results presented in Figure 11, as the fastener vertical stiffness increases from 25 kN/mm to 50 kN/mm, the deflection of the track structure, the fastener force and the disengagement length of the track slab are almost nonlinear monotonically. The upward (−14.5%) and downward (−0.4%) deflections of the rail and the upward (−48.1%) deflection of the track slab decrease with the increase of the fastener vertical stiffness, as shown in Figure 11a,b. On the contrary, the downward deflection of the track slab (0.3%), the pressure (19.5%) and pull (3.5%) of fasteners and the disengagement length of the track slab (2.3%) increase with the increase in the fastener vertical stiffness (Figure 11b–d). In conclusion, considering the nonlinear contact between layers, with the increase in the fastener vertical stiffness, the response of the track structure changes quickly at first and then slowly.

### 5.2. The Effects of the Elastic Modulus of CA Mortar Layer on Track Deformation

The mortar layer is located between the track slabs and concrete base plates, and plays an important role in supporting the track plate as well as buffering the impact of the high-speed train load. Therefore, the performance of the CA mortar will have a significant effect on the smoothness of the rail surface and train comfort and safety. The elastic modulus of mortar is typically between 100~300 MPa. AM-2 was used to calculate the mapping deformation of track structure and fastener force when the girder undergoes a vertical fault of 3 mm for five different elastic modulus of the mortar layer (100 MPa, 150 MPa, 200 MPa, 250 MPa, and 300 MPa). 

**Figure 12:**
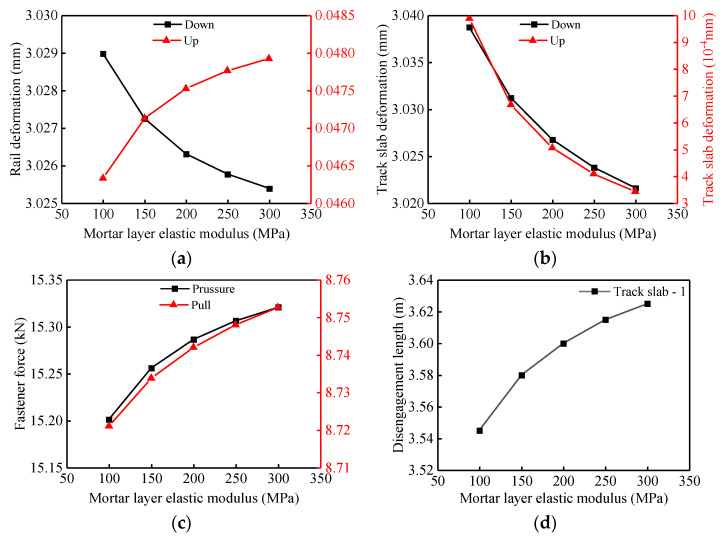
Effects of mortar layer elastic modulus on track structure: (**a**) variation of rail deformation; (**b**) variation of track slab deformation; (**c**) variation of fastener force extremum; (**d**) variation of track slab disengagement length.

According to the results presented in Figure 12, as the mortar layer elastic modulus increases from 100 MPa to 300 MPa, the deflection of the track structure, the fastener force and the disengagement length of the track slab are all nonlinear monotonically. The downward (−0.1%) deflection of the rail and the upward (−65.4%) and downward (−0.6%) deflections of the track slab decrease with the increase of the mortar layer elastic modulus, as shown in Figure 12a,b. On the contrary, the upward (3.5%) deflection of the rail, the pressure (0.8%) and pull (0.4%) of fasteners and the disengagement length of the track slab (2.3%) increase with the increase of the mortar layer elastic modulus (Figure 12a,c,d). In summary, considering the nonlinear contact between layers, with the increase of the mortar layer elastic modulus, the response of the track structure changes quickly at first and then slowly.

## 6. Conclusions

Based on the principle of stationary potential energy and considering the nonlinear contact between mortar layer and track slabs, a theoretical model for mapping the relationship between vertical deformation of the bridge structure and rail deformation was established. Solutions of the theoretical model were compared to the results of the finite element model and the experimental model. The influence of the amplitude of vertical structure deformation, the fastener vertical stiffness, and the mortar layer elastic modulus on the rail mapping deformation were analyzed. The main conclusions can be summarized as follows:

(1) Theoretical results of rail mapping deformation caused by various typical vertical deformations of the bridge structure agree well with the results of the FEM and the experiment model, which verifies the proposed theoretical model. When the mortar layer is considered as elastic and nonlinear, the rail mapping deformation is obviously different. The nonlinearity of the vertical stiffness of the mortar layer cannot be ignored.
(2) With the deformation of the bridge structure, the track structure has obvious following property. With the increase of the girder deflection, the upwarping deformation of the track structure shows an obvious nonlinear increasing trend due to the occurrence of the track slabs disengagement. The track slabs’ disengagement mainly occurs near the beam joints (the side of the deformed beam).
(3) When nonlinear contact between mortar layer and track slabs was considered, as the deflection amplitude of the girder increases, the forces of the fasteners and the disengagement length of the track slabs are obviously nonlinear.
(4) As the fastener vertical stiffness and/or the mortar layer elastic modulus increases the track deflection, the fastener force and the track slabs disengagement length are all nonlinear monotonically. The larger vertical stiffness of the fasteners and the elastic modulus of the mortar layer will have adverse effects on the life and safety of the track structure.

## Figures and Tables

**Table 1:** Parameters of the unit slab-type ballastless track-bridge system.

Structure	Parameters	Value	FEM	AM-1(AM-2)	Notes
Rail	Elastic modulus	2.060 × 10^5^ MPa	1	1	CHN60
	Density	8.005 × 10^3^ kg/m^3^	1	1	
	Cross-sectional area	7.705 × 10^−3^ m^2^	1	4	
	Moment of inertia	3.217 × 10^−5^ m^4^	1	4	
Fastener	Vertical stiffness	40 kN/mm	1	4/0.625	WJ-7
	Horizontal stiffness	7 kN/mm	1	--	
	Interval	0.625 m	1	Continuous	
Track slab	Elastic modulus	3.600 × 10^4^ MPa	1	1	C60 concrete; Girder end: 3.75 m
	Density	2.549 × 10^3^ kg/m^3^	1	1	
	Sectional dimension	2.4 × 0.19 m	1	2	
	Moment of inertia	1.372 × 10^−3^ m^4^	1	2	
	Length	5.000 m	1	1	
CA mortar layer	Elastic modulus (*E*)	200 MPa	1	1	Compression only; *k_v_* = *EA*/*h*, *A* = 1 × *b*.
	Sectional dimension (*b × h*)	2.4 × 0.05 m	1	2	
	Vertical stiffness (*k_v_*)	9.600 × 10^9^ N/m	1	2	
	Horizontal stiffness	9.600 × 10^8^ N/m	1	--	
Base plate	Elastic modulus	3.250 × 10^4^ MPa	1	--	C40 concrete; Assumption (d).
	Density	2.549 × 10^3^ kg/m^3^	1	--	
	Sectional dimension	2.8 × 0.26 m	1	--	
	Length	5.000 m	1	--	
Girder	Elastic modulus	3.450 × 10^4^ MPa	1	1	C50 concrete
	Density	2.549 × 10^3^ kg/m^3^	1	1	
	Moment of inertia	1.093 × 10^1^ m^4^	1	1	

Note: Parameters in columns “AM” and “FEM” are multiplied in the “Value” column.

**Table 2:** The maximum and minimum rail mapping deformation in the two cases.

Case	Components	AM-1		AM-2		FEM		The Error between AM-2 and FEM	
		+(mm)	−(mm)	+(mm)	−(mm)	+(mm)	−(mm)	+(%)	−(%)
1	Rail	0.335	−10.34	0.147	−10.13	0.154	−10.13	−4.55	0
	Track slab	0	−10.00	0	−10.10	0	−10.20	0	−0.98
2	Rail	0.325	−9.67	0.133	−8.68	0.139	−8.68	−4.32	0
	Track slab	0	−9.95	0	−8.94	0	−9.04	0	−1.11

## Data Availability

Data sharing not applicable.
